# Significance of next-generation sequencing in the era of targeted therapy for biliary tract cancer: an analysis at a single institution

**DOI:** 10.37349/etat.2026.1002402

**Published:** 2026-09-18

**Authors:** Kei Nakagawa, Hideaki Sato, Masahiro Iseki, Daisuke Douchi, Shuichi Aoki, Michiaki Unno

**Affiliations:** Tan Tock Seng Hospital, Singapore; ^1^Department of Surgery, Tohoku University Graduate School of Medicine, Sendai 980-8574, Japan; ^2^Division of Hepato-Biliary and Pancreatic Surgery, Tohoku Medical and Pharmaceutical University, Sendai 983-8536, Japan

**Keywords:** next-generation sequencing, biliary tract cancer, precision medicine, molecular target drugs, ESCAT, targeted therapy

## Abstract

**Aim:**

Treatment approaches for biliary tract cancer (BTC) vary widely. Genomic profiling through next-generation sequencing (NGS) has made it possible to apply precision medicine in the management of BTC. This retrospective observational study aimed to evaluate the implementation of treatments based on identified genomic mutations and their clinical significance in patients with BTC in Japan, where NGS is covered by health insurance.

**Methods:**

We conducted a retrospective analysis of 65 patients with unresectable or recurrent BTC who underwent comprehensive genomic profiling at a single institution between November 2019 and October 2024. Genomic mutations were classified according to the ESMO Scale for Clinical Actionability of molecular Targets (ESCAT) scale. Overall survival (OS) was estimated using the Kaplan-Meier method and compared using the log-rank test.

**Results:**

Among the 65 patients, clinically relevant genomic mutations classified using the ESCAT scale were identified in 47 patients, and clinically treatable genomic mutations were identified in 13 patients. Six patients ultimately received mutation-guided therapy, including fibroblast growth factor receptor inhibitors and pembrolizumab. The median time from specimen submission to review by the expert panel was 26 (range, 16–40) days. No significant difference in OS was observed based on the site of the primary tumor (log-rank test, *P* = 0.5804).

**Conclusions:**

Although clinically actionable genomic mutations were identified in one-fifth of patients, < 10% ultimately received treatment based on those findings, highlighting a gap between genomic profiling and treatment implementation. In contrast, with the increasing number of available therapies, access to precision treatment for patients with BTC may improve in the future through a combination of early adoption of NGS and the complementary use of liquid biopsy when tissue is unavailable.

## Introduction

Biliary tract cancer (BTC) is a rare malignant tumor with a poor prognosis. BTC encompasses a highly heterogeneous group of tumors arising in various anatomical locations within the biliary tract, including the intrahepatic bile ducts, perihilar region, distal extrahepatic bile ducts, gallbladder, and ampulla. These subtypes exhibit significant differences in biological characteristics, molecular features, and clinical management. Because BTC is a rare cancer, the approach taken to date in developing systemic therapies has been to treat it as a single disease encompassing multiple anatomical subtypes, rather than formulating site-specific treatment strategies. However, in recent years, accumulating clinical trials and translational insights have gradually reshaped the therapeutic landscape for both unresectable and recurrent diseases and adjuvant settings [1].

In unresectable or recurrent BTC, gemcitabine plus cisplatin (GC) has been the standard first-line chemotherapy since the ABC-02 trial demonstrated a survival benefit over gemcitabine alone [2]. More recently, the addition of S-1 to GC (GCS) has shown improved survival in East Asian patients and has become an additional first-line treatment option [3]. A major recent advancement has been the positive results of immune checkpoint inhibitors (ICIs) combined with chemotherapy. In the TOPAZ-1 study (a randomized, double-blind, placebo-controlled phase III study), durvalumab + GC provided proof-of-concept that checkpoint blockade may enhance survival in BTC (12.8 vs. 11.5 months, hazard ratio [HR] = 0.80, *P* = 0.0021) [4]. KEYNOTE-966 provides further confirmatory evidence and broadens the acceptance of immunochemotherapy in first-line settings (12.7 vs. 10.9 months, HR = 0.83, *P* = 0.0034) [5]. Despite recent therapeutic advances, effective treatment options beyond first-line therapy remain limited. Although FOLFOX and postoperative S-1 have improved outcomes in selected settings, further advances are expected to depend on precision medicine and molecularly targeted therapies [6–8].

In summary, although BTC comprises biologically and surgically diverse entities with historically limited treatment options, its clinical approach is gradually evolving. In unresectable or recurrent diseases, immunochemotherapy has now been validated by phase III evidence, and combinations with S-1 have expanded options in East Asia. In the postoperative setting, adjuvant S-1 is supported by a randomized trial, and investigational immunochemoradiation is emerging. Despite these advances, durable disease control remains limited for many patients, and treatment options beyond first-line therapy remain inadequate. Consequently, increasing attention has focused on precision oncology, in which molecular profiling is used to identify actionable genomic alterations and guide targeted therapies. Recent genomic studies have shown that there is significant molecular heterogeneity among BTC subtypes. For example, fibroblast growth factor receptor 2 (FGFR2) fusion and isocitrate dehydrogenase 1 (IDH1) mutations are primarily observed in intrahepatic cholangiocarcinoma (ICC), whereas human epidermal growth factor receptor 2 (HER2) amplification is more frequently detected in gallbladder cancer (GBC). These differences have a direct effect on treatment. This highlights the importance of comprehensive genomic profiling in the molecular stratification of BTCs and the selection of therapeutic approaches.

Against this backdrop, genomic analyses and the development of precision medicine have been pursued for BTC, similar to other carcinomas, leading to the development of drugs that target specific genomic alterations. Consequently, the efficacy of molecularly targeted drugs, such as FGFR inhibitors, has been demonstrated and has already been incorporated into clinical practice. Although this represents a breakthrough treatment with promising efficacy, precision medicine is inherently limited to specific targets in a diverse range of genetic abnormalities, resulting in a restricted pool of eligible cases. Accurate detection of actionable alterations through next-generation sequencing (NGS) has become essential for BTC management.

In Japan, cancer gene panel testing has been covered by public insurance since 2019. NGS is now covered by insurance for advanced solid cancers that have completed or are expected to complete standard treatment and for rare or primary unknown tumors lacking standard treatment options. An expert panel (molecular cancer board) interprets the results and deliberates on treatment eligibility, with a mandatory registration of all cases at the National Data Center (Center for Cancer Genomics and Advanced Therapeutics [C-CAT]) established by the government [9]. Under this system, even when NGS results are available, they may not be discussed with patients. Despite the increasing use of NGS in clinical practice, several important questions remain unresolved, including the optimal timing of testing, appropriate selection of tissue versus liquid biopsy, and the extent to which expert panel recommendations are translated into matched therapies in real-world practice.

For surgeons, improved outcomes in unresectable BTC require reconsideration of surgical indications, whereas the expanding availability of molecularly targeted therapies has important implications for the management of postoperative recurrence. As a high-volume referral center for BTC, the Department of Surgery at Tohoku University performs both complex surgical resection and systemic therapy for unresectable disease. Therefore, we conducted this retrospective study to evaluate the real-world implementation of NGS in patients with BTC treated at our institution. Specifically, we investigated the spectrum of genomic alterations, expert panel recommendations, implementation of matched therapies, and practical issues related to the timing of NGS and specimen selection, including tissue and liquid biopsies.

## Materials and methods

### Patients

The inclusion and exclusion criteria are shown in Table 1. All consecutive patients who met the inclusion criteria during the study period were included. Because of the retrospective observational design, no formal sample size calculation was performed.

**Table 1:** The inclusion and exclusion criteria.

**Types of criteria**	**Details of the content**
**Inclusion criteria**	• Histologically confirmed biliary tract cancer (intrahepatic cholangiocarcinoma, periportal cholangiocarcinoma, distal cholangiocarcinoma, gallbladder cancer, or ampullary cancer). • Underwent comprehensive genomic profiling by an expert panel between November 2019 and October 2024. • Clinical-pathological and follow-up data were available. • Both resectable and unresectable/recurrent cases were included.
**Exclusion criteria**	• The tumor specimen was unsuitable for NGS owing to insufficient tumor content. • Clinical-pathological information was incomplete.

This observational study retrospectively reviewed 65 BTC cases submitted for diagnosis by an expert panel from the Department of Surgery, Tohoku University, between November 2019 and October 2024. During this period, the patients treated in our department were as follows: 175 cases of resection (six cases of intrahepatic bile ducts [ICC], 66 cases of hilar bile duct [perihilar cholangiocarcinoma (PHC)], 39 cases of distal bile duct, 27 cases of gallbladder [GBC], and 37 cases of ampullary bile duct [Vater]). Furthermore, there were 84 cases of chemotherapy for inoperable or recurrent disease (20 cases of ICC, 33 cases of PHC, 9 cases of distal, 18 cases of GBC, and 4 cases of Vater).

These represent all the cases in which genetic testing was performed on specimens during this period. Only one case was outside the scope of insurance coverage, and the remaining 64 cases were covered by insurance. Cases that had already been NGS-analyzed at other institutions and referred for treatment purposes were excluded. Additionally, some specimens were deemed unsuitable for submission because of insufficient tumor content at the time of specimen preparation and were not included in this analysis. For non-resected cases, biopsy specimens or resected metastatic lesions were submitted for NGS. For cases with recurrence after resection, sections were prepared from appropriately preserved resected specimens and submitted for NGS. For cases where tissue could not be obtained, NGS was performed using blood samples.

Comprehensive genomic profiling was performed using FoundationOne^®^ CDx, FoundationOne^®^ Liquid CDx, or Guardant360^®^ CDx, according to clinical indications.

Expert panels are held regularly in each region and include not only the patient’s attending physician but also experts with specialized knowledge and skills in areas such as cancer chemotherapy, genetic medicine, genetic counseling, pathology, molecular diagnostics, cancer genomics, and bioinformatics necessary for genetic analysis using NGS. Based on test results, the panel evaluates the biological significance of detected genetic mutations, determines the availability of corresponding drugs, and prioritizes recommended treatments and clinical trials to identify the most appropriate treatment for each patient.

Cases were extracted from the database, and genomic alterations and treatment proposals based on the expert panel’s interpretations were compiled. All available follow-up data were confirmed in September 2025. The median observation period from the submission of NGS specimens was 553 days.

This study was conducted in accordance with the principles of the Declaration of Helsinki and was approved by the Ethics Committee of Tohoku University Graduate School of Medicine (2024-1-668).

### Statistical methods

Overall survival was estimated using the Kaplan-Meier method and compared using the log-rank test. Statistical analyses were performed using JMP^®^ Student Edition 18.2.2 (JMP Statistical Discovery LLC, SAS Institute Inc., Cary, NC, USA). A two-sided *P* value < 0.05 was considered statistically significant.

## Results

### Baseline characteristics

Patient age ranged from 33 to 83 (median, 67) years. Regarding sex distribution, 44 were men and 21 were women. The primary tumor sites were intrahepatic in 14, hilar in 22, distal in 7, gallbladder in 18, and papillary site of the ampulla of Vater in four patients (Table 2).

**Table 2:** Baseline characteristics of the study population.

**Variables**	***n* (%) or median (range)**	**Details**
Number of patients	65	
Age (years)	67 (33‒83)	Median (range)
Sex		
Male	44 (68%)	
Female	21 (32%)	
Primary tumor site		
Intrahepatic (ICC)	14 (22%)	
Perihilar (PHC)	22 (33%)	
Distal	7 (11%)	
Gallbladder (GBC)	18 (28%)	
Ampulla (Vater)	4 (6%)	
Disease status at molecular testing		
Initially unresectable	26 (40%)	Locally advanced: 8 (12%) Distant metastasis: 18 (28%)
Recurrence after resection	39 (60%)	
First-line chemotherapy regimen		
GC (gemcitabine + cisplatin)	13 (20%)	
GCS (gemcitabine + cisplatin + S-1)	34 (52%)	
GCD (gemcitabine + cisplatin + durvalumab)	11 (17%)	
GCP (gemcitabine + cisplatin + pembrolizumab)	2 (3%)	
Others	5 (8%)	
Number of patients	65	

ICC: intrahepatic cholangiocarcinoma; PHC: perihilar cholangiocarcinoma; GBC: gallbladder cancer.

Based on pathology, 26 cases were determined to be unresectable at the initial diagnosis or at the time of open surgery. Of these, eight cases were locally advanced, and 18 cases had distant metastases. Among these 18 cases, three were deemed unresectable after planning surgery and review via diagnostic laparoscopy, and seven were unresectable after open surgery. Additionally, 39 patients experienced recurrence after resection.

The first-line chemotherapy regimens selected for these patients were GC in 13, GCS in 34, gemcitabine + cisplatin + durvalumab in 11, and gemcitabine + cisplatin + pembrolizumab in two patients. Ten patients received second-line treatment at the time of NGS. At the time of NGS testing, 10 patients were receiving second-line therapy. Among these patients, only a subset ultimately received matched therapies based on expert panel recommendations.

Follow-up through October 2025 revealed 45 deaths from the primary disease, two deaths from other causes, and 18 patients were still alive.

### Status of NGS implementation

The types of tests implemented for NGS were FoundationOne^®^ CDx (F1) in 59 patients, FoundationOne^®^ Liquid (F1L) in three patients, and Guardant 360^®^ (G) in three patients (Table 3).

**Table 3:** Details of next-generation sequencing (NGS).

**Variables**	***n* (%)**
NGS platform used	
FoundationOne^®^ CDx (F1)	59 (90%)
FoundationOne^®^ Liquid (F1L)	3 (5%)
Guardant360^®^ (G)	3 (5%)
Sample type for F1 (*n* = 59)	
Primary resection specimen	32 (54%)
Primary tumor biopsy	13 (22%)
Metastatic resection specimen	13 (22%)
Metastatic tumor biopsy	1 (2%)

The median times from specimen collection to submission were 306 (range, 13–1,636) days for F1 and 3 (range, 0–4) days for F1L and G, which used blood samples. Among the 59 patients who underwent tissue-based F1 testing, the submitted specimens consisted of primary resection specimens (*n* = 32), primary biopsies (*n* = 13), metastatic resection specimens (*n* = 13), and metastatic biopsies (*n* = 1). The time from sample submission to expert panel review ranged from 16 to 40 (median, 26) days. Because expert panel meetings were held weekly, there was no significant difference in the turnaround time between tissue-based testing (median, 26 days; range, 16–40) and blood-based testing (median, 26 days; range, 19–40).

### NGS analysis results

#### Integration of NGS results into treatment

Thirteen (20%) patients were proposed for insurance-covered treatment or clinical trial participation by an expert panel (Table 4).

**Table 4:** ESCAT tier classification of actionable genes.

**Case no.**	**Primary site**	**Proposed treatment**	**Treated**	**Tier I-A**		**Tier I-B**	**Tier II**		**Tier III**				**Tier IV**		**Tier V**	**Tier X**
				***FGFR2***	***IDH1***	***MSI-H/dMMR***	***BRAF* ^V600E^**	***NTRK***	***HER2***	***BRCA1/2***	***PIK3CA***	***KRAS G12C***	***MET***	***EGFR***	***CTNNB1/WNT***	***TP53/CDKN2A/SMAD4***
1	Vater	Yes	FGFR-I	Yes												
2	Distal	Yes	FGFR-I	Yes												
3	ICC	Yes	FGFR-I	Yes												
4	ICC				Yes											Yes
5	ICC				Yes											
6	PHC	Yes	Pem			Yes			Yes							
7	PHC	Yes	Pem			Yes					Yes					
8	ICC	Yes	Pem			Yes										
9	PHC	Yes					Yes									Yes
10	GBC	Yes							Yes							Yes
11	GBC	Yes							Yes							Yes
12	GBC	Yes							Yes							Yes
13	GBC								Yes							Yes
14	GBC	Yes							Yes							
15	GBC	Yes							Yes							
16	PHC								Yes							
17	PHC									Yes						Yes
18	PHC									Yes						
19	PHC	Yes								Yes						
20	PHC										Yes					Yes
21	Distal															Yes
22	GBC															Yes
23	PHC															Yes
24	Vater															Yes
25	ICC															Yes
26	Distal															Yes
27	GBC															Yes
28	GBC															Yes
29	GBC															Yes
30	Distal															Yes
31	PHC															Yes
32	GBC															Yes
33	PHC															Yes
34	ICC															Yes
35	PHC															Yes
36	Vater															Yes
37	ICC															Yes
38	Distal															Yes
39	PHC															Yes
40	PHC															Yes
41	PHC															Yes
42	GBC															Yes
43	PHC															Yes
44	ICC															Yes
45	Vater															Yes
46	GBC															Yes
47	Distal															Yes

ESCAT Tiers I‒IV: clinically actionable genomic alterations according to tier; Tier I-A: approved in the same cancer type; Tier I-B: approved in other cancer types; Tiers II‒IV: experimental or emerging evidence; Tier V/X: low or no clinical actionability at present; FGFR-I: fibroblast growth factor receptor inhibitor; Pem: pembrolizumab.

Based on the primary site, these comprised two cases (14%) of ICC, four cases (18%) of hilar cholangiocarcinoma, one case (14%) of distal cholangiocarcinoma, five cases (28%) of GBC, and four cases (25%) of cancer of the papilla of Vater. However, these proposals included trials in which participation was difficult. Moreover, even when the same genetic alteration was identified, no treatment was proposed if no appropriate trial was available during the search period or if enrollment had already been closed. Moreover, as of October 2025, IDH1 remains unapproved for insurance-covered treatment in Japan, making it impossible to propose medications outside of clinical trials.

Treatment was administered in six (9%) patients, with a median age of 66 (range, 39‒80) years. Treatment with *FGFR* inhibitors was administered in three patients (one each for ICC, distal papilla, and Vater’s papilla) and pembrolizumab in three patients (one for ICC and two for PHC).

#### Genomic alterations identified using NGS

Cases with detected genomic alterations are summarized in Table 3, referencing the ESMO Scale for Clinical Actionability of Molecular Targets (ESCAT).

Tier I genomic alterations included *FGFR2* (*n* = 3), *IDH1* (*n* = 2), and microsatellite instability-high (MSI-H)/deficient mismatch repair (dMMR) (*n* = 3), totaling eight (12%) cases. Tier II genomic alterations included *BRAF*^V600E^ (*n* = 1, 2%). Tier III genomic alterations included *HER2* (*n* = 8) and BReast CAncer (*BRCA*) 1/2 (*n* = 3), totaling 11 (17%) cases. These genomic alterations have internationally available targeted therapies, potentially leading to effective treatments. The detection rate of these mutations was 31%.

### Comparison of survival rates

**Figure 1:**
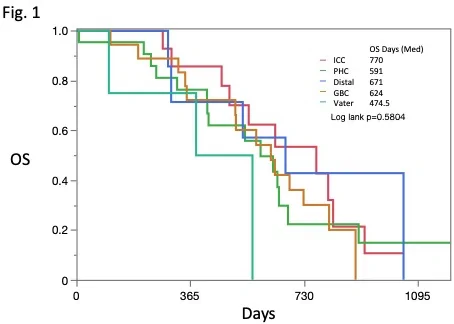
**Survival rate from the initiation of first-line treatment based on the primary site.** ICC: intrahepatic cholangiocarcinoma; PHC: perihilar cholangiocarcinoma; Distal: distal cholangiocarcinoma; GBC: gallbladder cancer; Vater: ampullary cancer.

In this study, we evaluated the overall survival from the start of first-line treatment according to primary tumor site. Although the prognosis for BTC generally varies depending on the anatomical site of origin, no significant difference in overall survival was observed between primary sites in this cohort (log-rank test, *P* = 0.5804) (Figure 1). This result may reflect the characteristics of the study population. The study population consisted of patients who had completed or were expected to complete standard treatment and were subsequently referred through a specialist panel to undergo comprehensive genomic profiling. Consequently, this cohort represented a highly selected group of patients with advanced or recurrent disease nearing the limits of standard treatment, which may have narrowed the survival differences between primary tumor sites.

**Figure 2:**
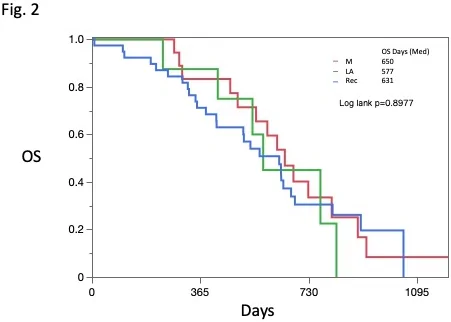
**Progression-state survival rate from the initiation of first-line treatment.** M: distant metastasis; LA: locally advanced; Rec: recurrence after resection.

Similarly, survival rates were compared according to distant metastasis (M), locally advanced disease (LA), and post-resection recurrence (Rec). No significant differences were observed between the groups (log-rank *P* = 0.8977) (Figure 2).

However, in cases of post-resection recurrence, approximately 10% of the patients died within 6 months of examination, possibly owing to a prolonged treatment history. This finding indicates that genetic testing may need to be initiated earlier in post-resection cases.

In this study, only six patients underwent NGS and were subsequently treated (Table 3). Statistically, with six versus 59 cases, the sample size ratio was 1:1, resulting in approximately 33.5% of the information content. This indicates limited statistical power, rendering the evaluation of survival curves via log-rank testing difficult.

**Figure 3:**
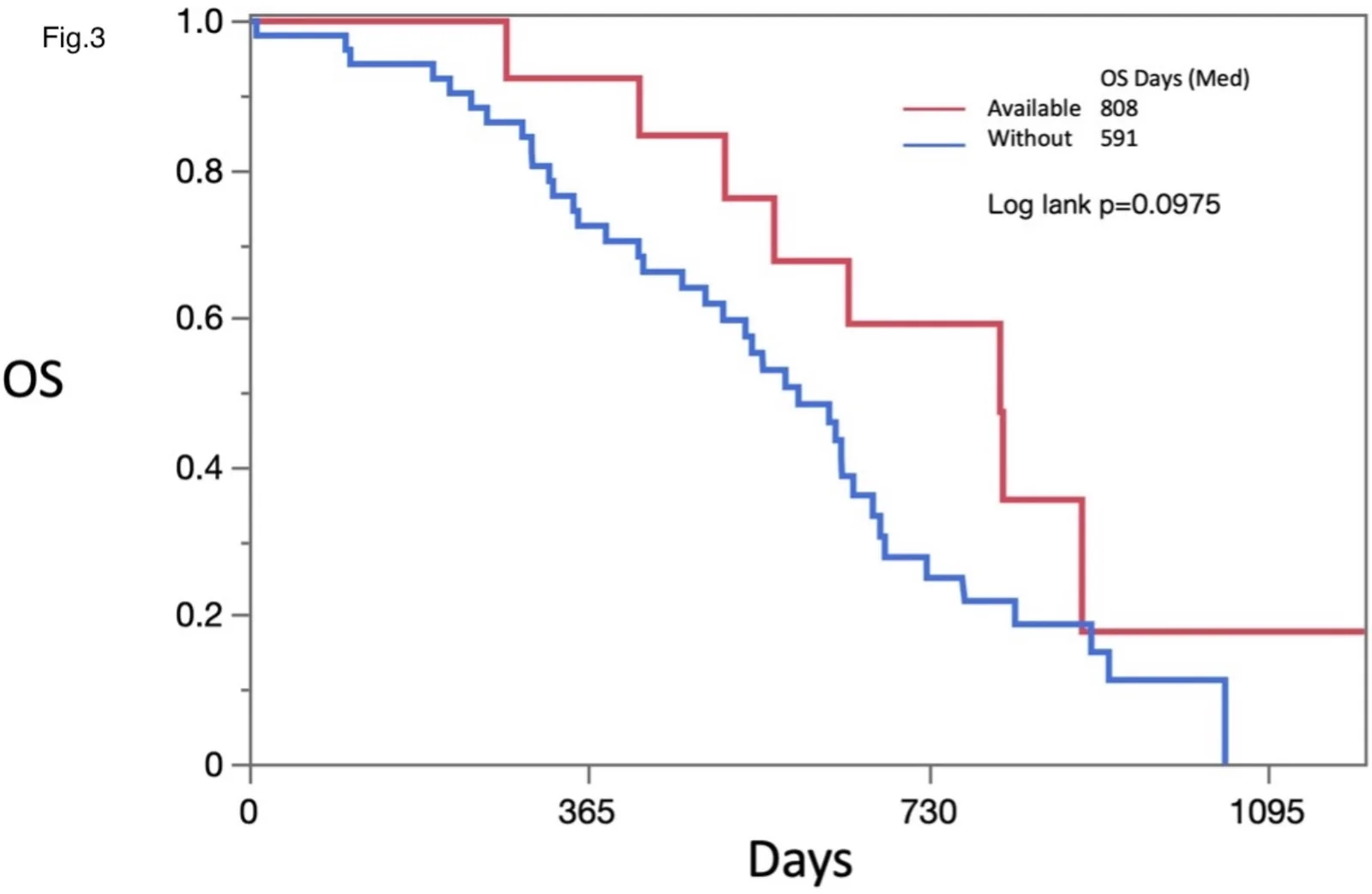
Survival rate depending on whether treatment proposals were made by the expert panel.

A comparison was conducted between the 13 cases for which treatment proposals were made by the expert panel and the others (Figure 3).

Moreover, because of the marked imbalance in the number of patients between the groups, meaningful statistical comparison was difficult. No significant difference in overall survival was observed between patients with and without expert panel treatment recommendations. Several prognostic factors were evaluated using Cox proportional hazards models (Table 5).

**Table 5:** Multivariate analysis of survival rates.

**Variables**	**Parameters (*n*)**	**Likelihood ratio *χ*^2^**	***P*-value (Prob > ChiSq)**
Proposed treatment	Available/Without	0.067	0.7956
Treatment based on NGS proposal	Available/Without	0.175	0.6755
First-line regimen	GC/GCS/GCD or GCP/others	8.703	0.1910
Disease status	M/LA/Rec	0.247	0.9696
Site	ICC/PHC/distal/GBC/Vater	5.110	0.2762
Tier-I alteration	Yes/No	0.647	0.4210
Tier-II alteration	Yes/No	3.240	0.0718

M: distant metastasis; LA: locally advanced disease; Rec: post-resection recurrence.

As this was an exploratory analysis of a heterogeneous cohort with different primary sites and treatment timings, the results demonstrated considerable variability. This analysis did not identify any significant factors influencing survival from the start of the first-line treatment.

## Discussion

In 2015, genomic sequencing studies of BTC revealed that approximately 40% of these cancers harbored genetic abnormalities that could serve as therapeutic targets [10]. This screening method is highly significant for BTC, for which promising treatment options beyond second-line therapy are scarce [11].

In Japan, cancer treatment is fundamentally covered by the national health insurance system, with diagnostic testing and therapeutic approval based on clinical evidence. Although this system has historically contributed to a drug lag, in which novel therapies approved overseas became available later in Japan [12], once approved, patients generally have access to these treatments with a defined level of personal cost sharing. However, in the era of precision oncology, the rapid development of molecularly targeted therapies presents new challenges in maintaining timely access to emerging treatments. Despite the requirement that comprehensive genomic profiling be performed after completion of standard therapy, insurance coverage for NGS has facilitated its clinical implementation in many patients. Furthermore, the nationwide accumulation of clinical, genomic, and prognostic data through the C-CAT provides an important resource for advancing precision medicine and generating real-world evidence in BTC.

The ESCAT is an assessment scale that classifies genomic alterations in cancer based on the efficacy of treatment with molecularly targeted drugs [13]. It is used as an indicator to aid treatment decision-making in the personalized treatment of various cancers [14]. In BTC, several genes are classified based on their drug discovery backgrounds. Tier I, which demonstrates significant efficacy in randomized and nonrandomized trials, includes *FGFR2* fusions, *IDH1* mutations, and MSI-H/dMMR. Tier II, which shows potential efficacy but requires further evidence, includes mutations that are useful in solid tumors, such as *BRAF*^V600E^ mutations and *NTRK* fusion gene abnormalities. Tier III, whose efficacy has been shown in other tumor types and is being validated in basket trials, includes *HER2* amplification/overexpression/mutation, *BRCA1/2* mutation, and *PIK3CA* mutation. Conversely, Tier X, which includes the *TP53/CDKN2A/SMAD4* mutation, has a high frequency, similar to that of pancreatic cancer, but lacks direct targeted therapies; these serve as prognostic markers or criteria for clinical trial inclusion.

These genetic alterations are presently significant for BTC, although their importance may evolve with drug development and validation.

In this study, *FGFR2* (*n* = 3), *IDH1* (*n* = 2), and MSI-H/dMMR (*n* = 3) were identified as Tier I. Owing to the progress of Japan’s insurance-covered treatments, only *FGFR*2 and MSI-H cases have led to actual drug administration.

FGFR is a receptor tyrosine kinase family comprising four members (FGFR1–4). The binding of fibroblast growth factors activates intracellular signaling pathways, including MAPK and PI3K/Akt/mTOR, thereby promoting cell proliferation [15]. Aberrant *FGFR* activation, through mutations, gene fusions, or amplifications, has been reported in multiple malignancies, including BTC. In cholangiocarcinoma, *FGFR2* alterations occur in approximately 5‒10% of cases [16, 17]. *FGFR2* fusions, particularly in ICC, drive alterations that promote tumor growth. Based on these findings, several *FGFR*-targeted agents have been developed and are now used clinically. Notably, *FGFR2* alterations in ICC are associated with a relatively favorable prognosis, indicating their potential utility as both predictive and prognostic biomarkers [18].

*IDH1* abnormalities are reported in approximately 10% of ICCs and 1% of extrahepatic cholangiocarcinomas [19]. In the United States, ivosidenib has been approved for the treatment of bile duct cancer with *IDH1* abnormalities. In Japan, this drug is under clinical investigation, and none of the patients in this study received it. In the near future, ivosidenib may be approved for insurance coverage as a treatment in Japan. They are likely to be introduced as a treatment option for a wide range of patient populations. The importance of NGS testing will increase.

MSI-H status is a key biomarker in precision oncology. MSI-H tumors arise from dMMR, leading to the accumulation of insertion-deletion errors in microsatellite regions during DNA replication, resulting in a hypermutated and highly immunogenic phenotype [20]. This results in a high mutational burden and the generation of tumor-specific neoantigens, which enhance T-cell recognition. Pembrolizumab, a programmed cell death protein 1 (PD-1) inhibitor, restores T-cell activity by blocking PD-1/programmed death-ligand 1 interactions and has demonstrated efficacy in MSI-H solid tumors. In Japan, pembrolizumab has been approved since 2018 for advanced or recurrent MSI-H solid tumors after the failure of standard chemotherapy. More recently, the addition of ICIs to gemcitabine and cisplatin has become the standard first-line treatment for BTC. In this context, although MSI-H/dMMR remains a predictive biomarker for immunotherapy response, its added clinical value may be limited when ICIs are already used in first-line treatment.

This study identified one case of *BRAF*^V600E^ as Tier II. *RAF* is one of the three genes that produce RAF proteins, which are involved in cell proliferation. Mutations in *BRAF*, such as V600E and V600K, have been observed and are believed to cause abnormal cell proliferation owing to the production of aberrant RAF proteins. *BRAF* abnormalities have been observed in malignant melanoma and lung cancer, and treatment with BRAF inhibitors has been approved for insurance coverage. *BRAF*^V600E^ solid tumors are estimated to account for < 1% of all solid tumors; however, they are found in 5–7% of BTCs and are particularly common in ICC, making them a promising treatment target [21]. The efficacy of combination therapy with the molecularly targeted drugs dabrafenib (a *BRAF* inhibitor) and trametinib (an *MEK* inhibitor) has been confirmed in unresectable advanced or recurrent solid tumors with *BRAF*^V600E^ mutation positivity. This combination therapy has been approved for insurance coverage in Japan. In this case, treatment was initiated before the introduction of this therapy in Japan, and medication was not administered.

In Tier III, genomic alterations were identified in 11 (17%) cases, comprising eight cases of *HER2* and three cases of *BRCA1/2*.

*HER2* (*ERBB2*) amplification or overexpression is observed in extrahepatic cholangiocarcinoma and GBC. We previously reported a high frequency of *HER2* positivity in GBC associated with pancreaticobiliary maljunction [22]. *HER2-*targeted therapies, including antibody-drug conjugates, such as trastuzumab deruxtecan, are currently being evaluated and have demonstrated promising activity. Although enrollment in clinical trials was theoretically possible in the present cohort, it was not practically feasible because of timing constraints and patient preference. Additionally, bispecific *HER2*-directed antibodies, such as zanidatamab, have shown encouraging efficacy in metastatic *HER2*-positive BTC and received accelerated approval by the US Food and Drug Administration in November 2024 [23]. In Japan, an application for the expanded indication of trastuzumab deruxtecan for *HER2-*positive solid tumors was submitted in April 2025, and its clinical implementation is anticipated. These developments highlight the importance of *HER2* as a therapeutic target in BTC.

*BRCA1/2* are involved in the DNA damage response. Mutations in these genes increase susceptibility to cancer. *BRCA* mutations elevate cancer risk not only for hereditary breast and ovarian cancer but also for gastric and pancreatic cancer and BTC [24]. Platinum-based anticancer drugs and poly ADP-ribose polymerase inhibitors may also be effective against bile duct cancers caused by *BRCA1/2* mutations.

A retrospective study of 327 cases examining ESCAT and treatment status in advanced BTC has been reported [25]. The median overall survival period was 22.6 months in patients with ESCAT I‒IV alterations who received matched treatment, which was higher compared with the 14.3 months in those without actionable ESCAT mutations (HR, 0.58; 95% confidence interval [CI], 0.40‒0.85; *P* = 0.005). Furthermore, among patients receiving matched targeted therapy, those with ESCAT I–II mutations had a longer median progression-free survival than those with ESCAT III–IV mutations (5.0 vs. 1.9 months; HR, 0.36; 95% CI, 0.15‒0.87; *P* = 0.02). In this study, it was difficult to evaluate whether the prognosis improved with treatment intervention in cases where NGS results led to new treatments, owing to the small number of cases. Visually, the Kaplan-Meier curves nearly overlapped. Notably, this analysis reflects the early phase of NGS implementation, when testing was often performed only after disease progression at the discretion of the attending physician. Only six of the 65 patients (9.2%) ultimately received matched therapies. Although numerous genomic mutations have been identified in BTC, the number of drugs with proven efficacy remains low. However, even during the course of this study, the development of potential therapeutic agents has continued to advance. The median overall survival period with drug therapy for BTC remains short, making targeted therapy crucial. We hope that in the future, more treatment options will be proposed, including basket trials that are not limited to specific cancer types.

Delays in proposing promising treatment options have led to more patients starting therapy under poorer conditions than those participating in clinical trials. This results in the loss of treatment opportunities and inadequate response rates. Many clinical trials of molecularly targeted drugs have shown promising outcomes. To achieve these results, it is crucial to avoid missing the window of treatment initiation. The median survival period for first-line treatment of BTC is generally only approximately 1.5 years. Even when specimens are prepared promptly, the median time from test submission to interpretation of the results is 26 days. Communicating the results to the patient and initiating a new treatment takes approximately 1 month. This timeframe is significantly long if the disease progresses without responding to standard therapy. It is necessary to consider securing specimens and performing NGS during standard treatment. When promising treatment options are identified, it is appropriate to shift promptly to the selected treatment once the standard therapy fails.

In the present study, gene identification using NGS was feasible even in resected specimens stored for > 4 years. In clinical practice, this finding suggests that archived surgical specimens can serve as a valuable resource for genomic analysis. However, careful handling of resected tissues remains essential to ensure the quality of genomic data. In particular, excessive formalin fixation and cross-contamination during sample processing should be avoided [26]. Furthermore, it is important to confirm the presence of sufficient tumor content at the time of pathological evaluation after resection, as inadequate tumor cellularity may compromise the success of genomic profiling.

Liquid biopsy represents an alternative approach when tumor tissue is difficult to obtain. This is particularly relevant in BTC, where biopsy samples are often limited in volume and quality. However, several limitations of liquid-based testing should be considered. First, the spectrum of detectable genomic alterations is narrower compared with tissue-based assays. Second, certain biomarkers, such as tumor mutational burden and germline mutations, cannot be adequately evaluated. Despite these limitations, liquid biopsy offers important advantages, including minimal invasiveness, ease of repeated sampling, and rapid turnaround time for mutation detection. Notably, in advanced disease, liquid biopsy achieves higher success rates for genomic profiling compared with tissue-based testing [27]. However, in Japan, logistical factors, such as sample transport and the scheduling of expert panel discussions, may prolong the time required for clinical decision-making, thereby partially offsetting the inherent speed advantage of liquid-based approaches. Thus, the optimal timing and modality of genomic testing should be determined on a case-by-case basis, considering disease status and sample availability.

The therapeutic landscape of BTC has recently evolved with the introduction of ICIs in combination with chemotherapy. Although a subset of patients achieves durable responses, only a limited proportion derive long-term benefit. In this context, NGS-guided treatment strategies are expected to play an increasingly important role, particularly in the second-line setting. Targeted therapies aimed at specific genomic alterations have demonstrated clinical benefit, but in routine practice, only a small proportion of patients receive these treatments.

Importantly, recent advances in molecularly targeted therapies have heightened the clinical significance of genomic profiling. In Japan, trastuzumab deruxtecan is expected to become available for *HER2*-positive solid tumors, including BTC. Similarly, IDH1 inhibitors are expected to become available for use in patients with *IDH1*-mutant advanced BTC. These developments underscore the growing importance of identifying treatable genomic alterations at earlier stages of the disease. Based on the findings of this study, two cases of IDH1 and seven cases of HER2 (nine cases, 14%) that did not receive drug treatment during this study may be included in the treatment group. As more targeted therapies become clinically available, the timing of NGS testing is likely to shift to earlier stages of treatment.

Despite these advances, several challenges remain. The overall frequency of actionable mutations in BTC is relatively low, and access to targeted therapies or clinical trials remains limited in many cases. Furthermore, the integration of genomic information into routine clinical decision-making requires multidisciplinary collaboration, including molecular tumor boards or expert panels. In Japan, nationwide initiatives, such as the C-CAT system, have facilitated the interpretation of genomic data; however, further efforts are warranted to standardize recommendations and improve access to matched therapies.

From a surgical perspective, the role of NGS is particularly relevant in the management of postoperative recurrence. Early identification of actionable mutations during the initial treatment phase may allow timely initiation of targeted therapies at recurrence. Therefore, when adequate tissue is available, early genomic profiling during primary treatment should be considered. In cases such as GBC, where obtaining tissue is often difficult, liquid biopsy can serve as a complementary approach, especially in advanced or recurrent disease.

This study has some limitations. First, this was a retrospective, single-center study with a relatively small sample size, which limited the statistical power and generalizability of the findings. Although multivariate survival analyses were performed, the relatively limited number of patients and outcome events precluded the inclusion of a larger number of clinically relevant covariates, such as performance status, comorbidities, and detailed treatment history, because doing so would have increased the risk of model overfitting and unstable estimates. Second, selection bias may have been introduced because only patients referred for comprehensive genomic profiling through the expert panel were included. Furthermore, under the current Japanese insurance system, comprehensive genomic profiling is generally performed after completion or expected completion of standard treatment, introducing the potential for survival bias by preferentially including patients who survived long enough to undergo NGS. Third, the study population was clinically heterogeneous, comprising patients with initially unresectable disease, locally advanced disease, and postoperative recurrence, and patients received different first-line treatment regimens. In addition, comprehensive genomic profiling was performed using different commercially available platforms (FoundationOne^®^ CDx, FoundationOne^®^ Liquid CDx, and Guardant360^®^ CDx), which may have introduced variability in the detection of genomic alterations despite their clinical validation. Fourth, because the primary objective of this study was to evaluate the real-world implementation of NGS in routine clinical practice, it was descriptive rather than comparative and was not designed to directly compare the optimal timing of NGS testing, tissue versus liquid biopsy, or different treatment strategies. Moreover, although clinically actionable genomic alterations were identified in one-fifth of the patients, only six ultimately received matched therapies because of limited drug availability, clinical trial accessibility, and regulatory restrictions in Japan, precluding a robust evaluation of the survival benefit of precision medicine. Fifth, we did not perform a formal health economic analysis; therefore, the cost-effectiveness of comprehensive genomic profiling could not be evaluated. In addition, although follow-up data were available through September 2025, longer-term outcomes require further investigation. Finally, the findings of this study reflect the current Japanese healthcare system, including insurance coverage and expert panel-based decision-making, and therefore may not be directly generalizable to healthcare systems in other countries. Nevertheless, this study provides valuable real-world evidence regarding the implementation of comprehensive genomic profiling, the gap between the identification of clinically actionable genomic alterations and the actual implementation of matched therapies, and practical considerations for optimizing the clinical use of NGS in BTC.

### Conclusion

In conclusion, this single-center retrospective study demonstrated that clinically actionable genomic alterations were identified in approximately one-fifth of patients with BTC; however, < 10% ultimately received matched therapies. These findings highlight a substantial gap between the identification of actionable genomic alterations and the implementation of precision medicine in routine clinical practice. Although NGS-based genomic profiling using archived surgical specimens and liquid biopsy was feasible, the clinical benefit of genomic-guided therapy could not be demonstrated in this study because of the limited number of patients who received matched treatment. As the availability of molecularly targeted therapies, including HER2-directed therapies and IDH1 inhibitors, continues to expand, the clinical value of comprehensive genomic profiling is expected to increase. Future prospective multicenter studies are required not only to optimize the timing of genomic profiling but also to bridge the gap between the identification of actionable genomic alterations and the actual implementation of matched therapies in patients with BTC.

## Abbreviations

BRCA: BReast CAncer
BTC: biliary tract cancer
C-CAT: Center for Cancer Genomics and Advanced Therapeutics
CI: confidence interval
dMMR: deficient mismatch repair
ESCAT: ESMO Scale for Clinical Actionability of molecular Targets
FGFR2: fibroblast growth factor receptor 2
GBC: gallbladder cancer
HER2: human epidermal growth factor receptor 2
HR: hazard ratio
ICC: intrahepatic cholangiocarcinoma
ICIs: immune checkpoint inhibitors
IDH1: isocitrate dehydrogenase 1
MSI-H: microsatellite instability-high
NGS: next-generation sequencing
PHC: perihilar cholangiocarcinoma

## Declarations

### Availability of data and materials

The datasets for this manuscript are not publicly available because they contain potentially identifiable genomic and clinical patient information and are subject to institutional ethics and privacy restrictions. Requests for accessing the datasets should be directed to Kei Nakagawa (nakagawa.kei@tohoku-mpu.ac.jp).

### Copyright

© The Author(s) 2026.
